# Comparison of the Gonial Angle With Age and Gender Using Cone-Beam Computed Tomography Images

**DOI:** 10.7759/cureus.24997

**Published:** 2022-05-14

**Authors:** Ayşe Bakan, Piraye Kervancıoğlu, İlhan Bahşi, Eda Didem Yalçın

**Affiliations:** 1 Anatomy, Gaziantep University, Gaziantep, TUR; 2 Dentomaxillofacial Radiology, Dokuz Eylül University, İzmir, TUR

**Keywords:** cone-beam computed tomography, dental anthropology, mandible, forensic anthropology, gonial angle

## Abstract

Introduction

The mandible is one of the most important bones used in gender determination in forensic medicine and anthropology. In literature, there are many studies examining the relationship between the gonial angle on the mandible and gender. However, these studies reported different results. This study aimed to measure the gonial angle with cone-beam computed tomography (CBCT) images and investigate the relation of this angle with age and gender.

Materials and methods

CBCT images of 235 dentate individuals (111 males and 124 females) aged between seven and 77 years were evaluated. The individuals examined were categorized into four age groups: 7-19 years (group I), 20-39 years (group II), 40-59 years (group III), and 60-77 years (group IV). The gonial angle was measured bilaterally in all individuals.

Results

The mean age of the males was 41.70 ± 19.14, and the mean age of the females was 39.47 ± 17.90 years. There was no statistically significant difference between the ages based on gender (p = 0.356). It was observed that there was a statistical difference between the gonial angle and gender in groups II and III. There was no correlation between age and gonial angle in all groups.

Conclusion

The results obtained in this study and the comparison of these results with the literature clearly show that it is currently not possible to clearly express the relationship between the gonial angle and both age and gender. For this reason, we believe that conducting further studies evaluating both the gonial angle and the relationship between the gonial angle and other anatomical structures on a larger sample can yield more meaningful results.

## Introduction

Many anatomical formations in the mandible such as the gonial angle, bigonial breadth, the height of mandibular ramus, antegonial angle, and antegonial depth can be used for gender estimation [1]. When examining human remains, accurate evaluation of the gonial angle values is important to determine gender, age, and dental status [2]. Furthermore, the gonial angle is particularly important in orthodontic research to understand the changes in the developmental period [3]. The gonial angle is associated with the function and shape of the muscles of mastication. Strong masseter and anterior temporal muscle activity are associated with a small gonial angle [4].

Due to this importance, the gonial angle has been studied in far too many studies [1-3,5-28]. In the previous studies, the gonial angle was generally compared with age [2,5-14,23] and gender [1,2,5-10,12-19, 23]. On the other hand, it has been reported that it is controversial whether there are gender and age differences in the gonial angle values [2,17]. There are also studies in the literature investigating the relation of the gonial angle with the dental status [5,7,9,20], the postoperative stability in the treatment of mandibular prognathism [29], the dynamic tongue collapse in children with snoring [30], the course of the inferior alveolar canal [28], osteoporosis among postmenopausal women [31], location of lingula [32] and different skeletal malocclusion types [33].

In these studies, various methods such as cone-beam computed tomography (CBCT) [1], multidetector computed tomography (MDCT) [8], panoramic radiography [2,6,10,12-15,19-23,26], cephalogram [5,16], both panoramic radiography and lateral cephalogram [25,27], both direct anthropometry and lateral cephalometry [24], and both CBCT and direct measurement of the dry bones [28] were used. It is known that the age, gender, and ethnicity of the studied group are not always recognized in studies with dry bones [34]. Additionally, CBCT has been accepted as an ideal imaging method for dentomaxillofacial diagnosis due to its low cost and low radiation dose [35,36]. This study aimed to measure the gonial angle with CBCT images and investigate the relation of this angle with age and gender.

## Materials and methods

**Figure 1:**
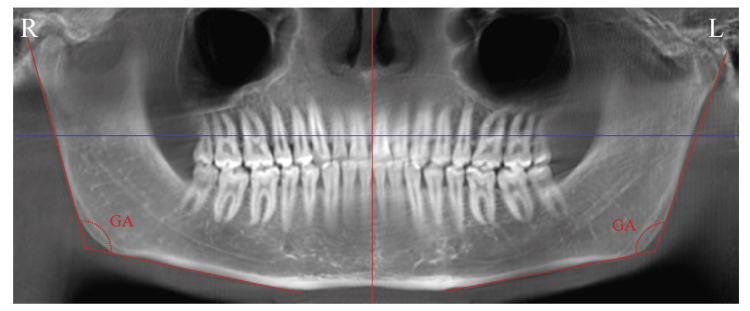
Measurement of the gonial angle on CBCT images CBCT: Cone-beam computed tomography; GA: Gonial angle; R: Right; L: Left.

Before the study was initiated, approval was obtained from the Ethics Committee of Gaziantep University (approval date: 05/02/2020 and approval number: 2020/60). The dentate individuals were examined, and those who had a congenital malformation, maxillofacial deformity, and a story of a craniofacial operation or trauma were excluded from the study. CBCT images of 235 individuals (111 males, 124 females, aged between seven and 77 years) who were admitted to Gaziantep University Faculty of Dentistry for any reason were evaluated; the CBCT images were obtained by the Planmeca Promax 3D scanner (Planmeca, Helsinki, Finland) on multiplanar sections in a standard resolution mode, voxel size: 0.4 mm^3^, and 16 cm × 9 cm, 16 cm × 16 cm field of view (FOV). The CBCT images were evaluated by Planmeca Romexis Viewer (Planmeca, Helsinki, Finland). As a result of examining the multiplanar images, measurements were made on the panoramic reformatted image. The individuals examined were categorized into four age groups: 7-19 years (group I), 20-39 years (group II), 40-59 years (group III), and 60-77 years (group IV). The gonial angle was measured bilaterally in all individuals with the Planmeca Romexis Viewer (Figure 1).

Statistical analysis

The data were evaluated statistically. The suitability of the data for normal distribution was tested using the Shapiro-Wilk test. The student’s paired t-test was used to examine the differences in the gonial angle between the genders. Pearson’s correlation coefﬁcient was calculated to estimate the relationship between the gonial angle and age. Also, one-way ANOVA (analysis of variance) was performed, when the variances were homogeneous, to observe the differences between the four age groups. Statistical Package for the Social Sciences (SPSS) v22.0 software (IBM Corp., Armonk, NY) was used for all analyses. A p-value < 0.05 was considered statistically significant.

## Results

The mean age of the males was 41.70 ± 19.14 years, and the mean age of the females was 39.47 ± 17.90 years. There was no statistically significant difference between the ages based on gender (p = 0.356). Descriptive statistics and comparison by gender for gonial angle values are presented in Table 1.

**Table 1:** The average values of the gonial angle by the groups and comparisons of these values by gender * indicates significant difference (p < 0.005). S.D.: Significant difference; M: Male; F: Female; R: Right; L: Left; n: Number of individuals.

	n		Right Gonial Angle ± S.D. (°)			Left Gonial Angle ± S.D. (°)		
	F	M	F	M	p-value	F	M	p-value
Group I	25	23	125.85 ± 7.56	124.45 ± 8.05	0.535	125.50 ± 7.45	124.55 ± 7.98	0.671
Group II	28	22	125.83 ± 7.87	120.27 ± 6.15	0.009*	125.70 ± 8.09	120.83 ± 6.04	0.023*
Group III	53	46	124.82 ± 5.83	120.66 ± 6.22	0.001*	124.02 ± 5.55	120.82 ± 6.91	0.012*
Group IV	18	20	124.81 ± 5.91	122.18 ± 7.16	0.227	123.66 ± 6.81	120.97 ± 7.01	0.240
Total	124	111	125.25 ± 6.61	121.64 ± 6.88	0.001*	124.62 ± 6.69	121.62 ± 7.07	0.001*

Homogeneity was found between all groups (right p = 0.565 and left p = 0.251 for females, and right p = 0.601 and left p = 0.393 for males). Additionally, it was found that there was no significant difference between the groups tested by one-way ANOVA (right p = 0.870 and left p = 0.591 for females, and right p = 0.123, and left p = 0.172 for males). There was no correlation between the age and gonial angle in all groups (right p = 0.164 and left p = 0.065 for females, and right p = 0.221 and left p = 0.073 for males).

## Discussion

Over a lifetime, the mandible is subjected to significant biomechanical stresses and development [17]. The mandible undergoes remodeling and morphological changes throughout an individual’s life [25]. Besides, the effect of muscle connections on the size and shape of the mandible is observed from the development of the mandible [37]. The constant forces exerted by the muscles and age-related changes in these forces have a great relationship with the morphological evolution of the mandible [38]. Age and gender can affect the shape of the mandible as the strength of these muscles can vary with age and gender. For these reasons, it is thought that the gonial angle on the mandible may also differ according to age and gender. Moreover, the gonial angle at the junction of the ramus and the body of the mandible defines the mandible’s shape and form. It is an essential parameter in evaluating the symmetry of the facial skeleton [25].

The skull and the pelvis are primary areas for gender determination in forensic anthropology [17]. Although the pelvis gives more reliable results in determining sex, cranium bones can also be used in gender determination [39,40]. Besides, the examination of more than one anatomical structure provides higher accuracy in gender prediction [17,39]. Foramen magnum and mandible from the significant formations within the cranium bones are among the structures used in gender prediction [39-41]. However, in forensic anthropology, skeletal remains may be encountered in some cases, rather than the whole body. Additionally, the mandible is the largest and most powerful part of the skull, and its identification is essential in forensic cases and anthropological studies, especially when examining skeletal remains [17,42]. Moreover, it is known that the mandible has a high degree of sexually dimorphic characteristics [1].

Therefore, in previous studies, many structures on the mandible such as bigonial breadth, the height of mandibular ramus, gonial angle, antegonial angle, and antegonial depth were examined for sex and age determinations [1,8-10,16]. Although evaluating more than one structure together provides more meaningful results, in some cases, it may be necessary to evaluate only a single structure such as the gonial angle in the skeletal remains. Therefore, in the literature, many studies examined the gonial angle according to age and gender [1,2,6,8-10,12,15-17,19,24,26,37,43]. However, the issue of whether there is a difference in the gonial angle by gender and age is controversial.

Relationship between gonial angle and gender

Ohm and Silness [5], Upadhyay et al. [24], Kanya et al. [15], and Dutra et al. [26] stated that the gonial angle did not differ statistically according to gender. In contrast, Chole et al. [9], Abu-Taleb and El Beshlawy [10], Belaldavar et al. [16], Huumonen et al. [43], Direk et al. [8], Jambunath et al. [19], Leversha et al. [6], and Shah et al. [37] reported that the gonial angle differs statistically according to gender and had lower value in males. Besides, Gamba et al. [1] and Bhuyan et al. [12] stated a difference between the gonial angle and gender, but they found lower values in females. Bulut et al. [17] examined the difference between the gonial angle and gender by categorizing the ages of the cases due to these differences in the literature and demonstrated that females have general larger gonial angles in all groups and that there are significant differences between males and females only in senior adults (60-80 years).

In our study, although this angle was found to be higher in females in all groups, it was observed that there was a statistical difference only in the groups between the ages of 20-60 years, in contrast to Bulut et al. [17] The differences in these studies show that the gonial angle alone may not be useful in determining the sex, just as Bulut et al. [17] stated. Perhaps, it may be useful to examine and compare the gonial angle with other anatomical structures. Therefore, evaluating the gonial angle and other anatomical structures in the skull bones together in larger groups will provide more beneficial results.

Relationship between the gonial angle and age

Shah et al. [37] stated that there was a statistically significant difference between the age and gonial angle. Direk et al. [8] reported that there was no significant difference in gonial angle according to the age groups. However, Shah et al. [37] and Direk et al. [8] did not indicate whether there is a correlation between the age and gonial angle. Larrazabal-Moron and Sanchis-Gimeno [2] reported that there was a significant negative correlation between the age and gonial angle. In contrast, Fattah and Hassan [7] said that the gonial angle increased with age. Chole et al. [9], Abu-Taleb and El Beshlawy [10], and Dutra et al. [26] reported that there was no correlation between the age and gonial angle. Bhuyan et al. [12] reported that the gonial angle increased on the left side in males with age, but there was no correlation on the right side in males and both sides in females. Upadhyay et al. [24] reported that there is a decrease in gonial angle with advancing age, but no significant pattern was observed in the analysis between groups. In this study, when the sides and genders were examined separately, it was observed that there was no correlation between the age and gonial angle. These results clearly show that it is very difficult to express a precise relationship between the age and gonial angle, just as between the gender and gonial angle.

Limitation

Due to the retrospective nature of this study, the lack of precise information about the ethnicity of the cases examined is the most important limitation of this study.

## Conclusions

Determining age and gender by examining human bones is extremely important, especially in forensic medicine and anthropology. Although it is controversial in the literature whether knowing only the gonial angle can be used for this purpose, the results obtained in this study and the comparison of these results with the literature clearly show that it is currently not possible to clearly express the relationship between the gonial angle and both age and gender. For this reason, we believe that conducting further studies evaluating both the gonial angle and the relationship between the gonial angle and other anatomical structures on a larger sample can yield more meaningful results.
